# Experimental strategies for the identification and characterization of adhesive proteins in animals: a review

**DOI:** 10.1098/rsfs.2014.0064

**Published:** 2015-02-06

**Authors:** Elise Hennebert, Barbara Maldonado, Peter Ladurner, Patrick Flammang, Romana Santos

**Affiliations:** 1 Biology of Marine Organisms and Biomimetics, Research Institute for Biosciences, University of Mons, 23 Place du Parc, 7000 Mons, Belgium; 2 Molecular Biology and Genetic Engineering, GIGA-R, University of Liège, 1 Avenue de l'Hôpital, 4000 Liège, Belgium; 3 Institute of Zoology and Center of Molecular Bioscience Innsbruck, University of Innsbruck, Technikerstrasse 25, 6020 Innsbruck, Austria; 4 Unidade de Investigação em Ciências Orais e Biomédicas, Faculdade de Medicina Dentária, Universidade de Lisboa, Cidade Universitária, 1649-003 Lisboa, Portugal

**Keywords:** biological adhesion, metazoans, protein characterization

## Abstract

Adhesive secretions occur in both aquatic and terrestrial animals, in which they perform diverse functions. Biological adhesives can therefore be remarkably complex and involve a large range of components with different functions and interactions. However, being mainly protein based, biological adhesives can be characterized by classical molecular methods. This review compiles experimental strategies that were successfully used to identify, characterize and obtain the full-length sequence of adhesive proteins from nine biological models: echinoderms, barnacles, tubeworms, mussels, sticklebacks, slugs, velvet worms, spiders and ticks. A brief description and practical examples are given for a variety of tools used to study adhesive molecules at different levels from genes to secreted proteins. In most studies, proteins, extracted from secreted materials or from adhesive organs, are analysed for the presence of post-translational modifications and submitted to peptide sequencing. The peptide sequences are then used directly for a BLAST search in genomic or transcriptomic databases, or to design degenerate primers to perform RT-PCR, both allowing the recovery of the sequence of the cDNA coding for the investigated protein. These sequences can then be used for functional validation and recombinant production. In recent years, the dual proteomic and transcriptomic approach has emerged as the best way leading to the identification of novel adhesive proteins and retrieval of their complete sequences.

## 1. Introduction

Biological attachment systems can be subdivided into several groups according to the fundamental physical mechanisms underlying their operation [1]. There are many systems based entirely on mechanical principles (e.g. hooks, suckers or friction devices), whereas others rely on the chemistry of polymers and colloids (diverse types of adhesives) [2]. Biological adhesives, also comprising glues and cements, can be defined as sticky materials preventing the separation of two substrates. The surface properties of the substrates and the chemical and physical properties of the adhesive determine the strength of adhesion and cohesion [3]. Adhesives present several advantages compared to other attachment systems: (i) they are versatile, being able to bind surfaces with various chemistry and roughness, (ii) they can join dissimilar materials, and (iii) they show improved stress distribution in the joint [3]. This is why adhesive systems are ubiquitous in nature, being found in bacteria, fungi, protists, plants and animals. Although the diversity of adhesion mechanisms in animals is huge, currently little is known of this complex phenomenon. However, in recent years, an increasing number of reports have been published on the characterization of biological adhesives [4–6].

In this review, we will focus on adhesives from animals, playing a key role in diverse functions and therefore varying widely in terms of structure, composition and capability. Adhesive systems occur in both aquatic and terrestrial animals. It is clear that the physico-chemical conditions are different underwater than in air, and therefore different kinds of adhesives will be required. However, all animal adhesive secretions are predominantly made up of proteins, although other components can be involved [4,5]. Recently, the development of new techniques allowing high-throughput characterization of protein-based materials, such as transcriptomics and proteomics, has accelerated the discovery of new adhesive proteins [7]. The aim of this review is to compile a list of the molecular methods that have been used to identify and characterize adhesive proteins in multicellular eukaryotic organisms (i.e. metazoans).

## 2. Animal protein-based adhesives

### 2.1. Diversity

From the biologists' point of view, adhesives may serve a variety of functions in metazoans: (i) attachment of an organism to a non-living surface, including dynamic attachment during locomotion and permanent fixation, (ii) attachment of one organism to another (phoresy or parasitism, prey capture), or (iii) attachment of exogenous materials together for the building of tubes, nests or burrows [6]. The evolutionary background and the biology of species on the one hand, and environmental constraints on the other hand, both influence the specific composition and mode of operation of the adhesive secretion in a particular organism. Biological adhesives can therefore be remarkably complex and involve a large range of interactions and components with different functions [4,5].

**Figure 1.**
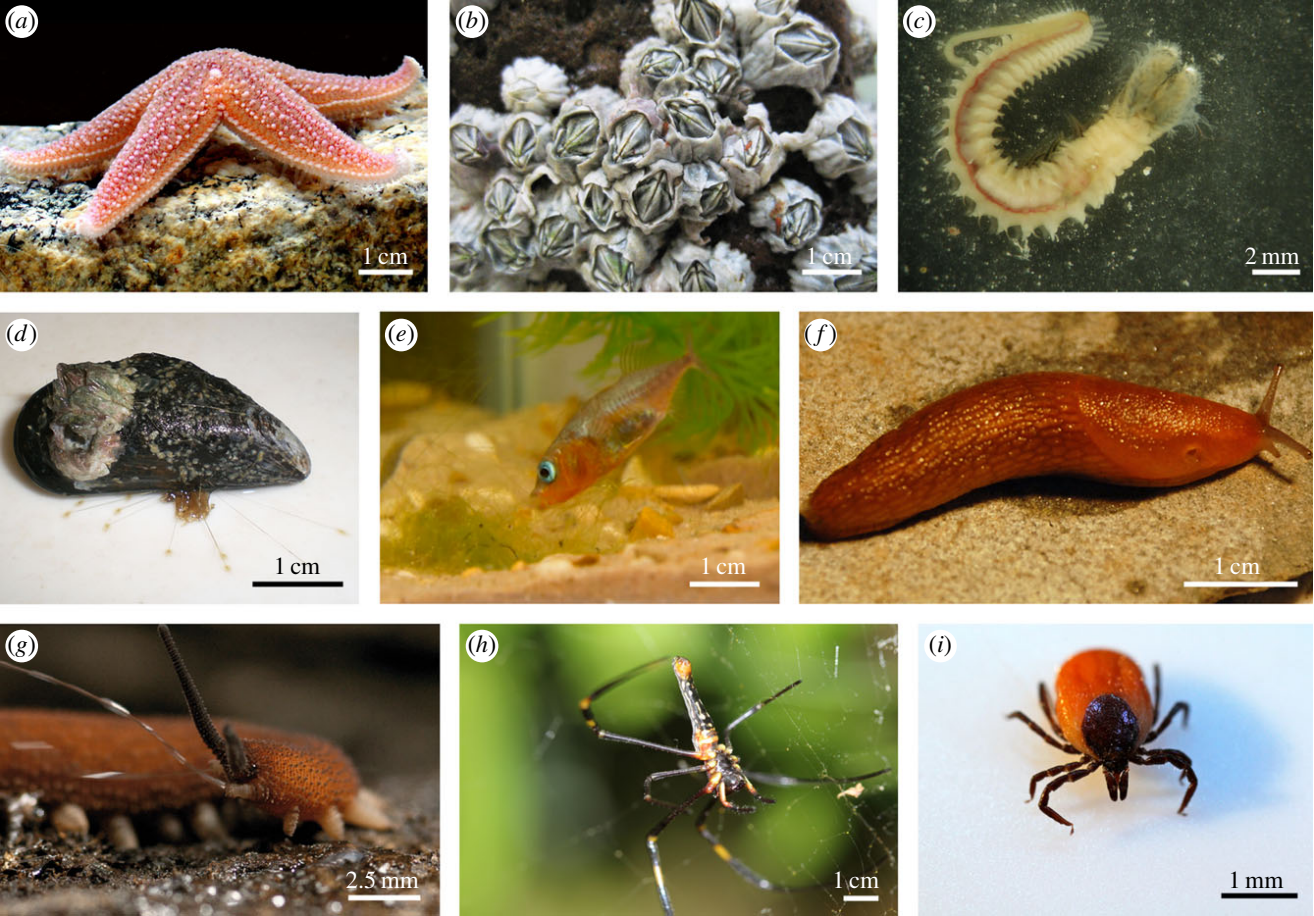
Model animals used for the study of biological adhesives. (*a*) Sea star of the species *Asterias rubens* attached to a rock by its tube feet. (*b*) Group of barnacles of the species *Elminius modestus* attached on a rock (picture courtesy of N. Aldred, Newcastle University, UK). (*c*) Polychaete of the species *Sabellaria alveolata* extracted from its tube. (*d*) Mussel of the species *Mytilus edulis* attached to a Teflon surface by means of byssal threads. (*e*) Three-spine stickleback of the species *Gasterosteus aculeatus* assembling its nest (picture courtesy of I. Barber, University of Leicester, UK). (*f*) Slug from the species *Arion fasciatus* creeping on a rock on an adhesive mucus film (picture courtesy of A. Smith, Ithaca College, USA). (*g*) Velvet worm of the species *Principapillatus hitoyensis* ejecting sticky threads for defence or prey capture (picture courtesy of A. Bär, University of Leipzig, Germany). (*h*) Spider of the species *Nephila pilipes* on its web (picture courtesy of J. Delroisse, University of Mons, Belgium). (*i*) Tick of the genus *Ixodes* (picture courtesy of J. Delroisse, University of Mons, Belgium). (Online version in colour.)

We have selected nine biological models (adult animals only) in which the complete sequence of at least one adhesive protein is known (a list of the proteins described in this review is presented in table 1). These organisms (figure 1) encompass the whole metazoan phylogeny, cover all types of habitats (marine, freshwater and terrestrial) and use protein-based adhesives for a large variety of functions (attachment, locomotion, prey capture, building and defence).

**Table 1.** List of the adhesive proteins cited in the text for which full-length sequences have been deposited in the National Center for Biotechnology Information.

common name	classification		species	protein	NCBI accession number	reference
sea star	Echinodermata	Asteroidea	*Asterias rubens*	Sfp-1	AHN92641	[8]
stickleback	Chordata	Actinopterygii	*Gasterosteus aculeatus*	spiggin	NM_001267690	[9]
velvet worm	Onychophora	Udeonychophora	*Euperipatoides rowelli*	Er_P1	HM217027	[10]
				Er_P2a	HM217028	[10]
				Er_P2b	HM217029	[10]
				Er_P3	HM217030	[10]
barnacle	Arthtropoda	Crustacea	*Megabalanus rosa*	Mrcp-19k	BAE94409	[11]
				Mrcp-20k	BAB18762	[12]
				Mrcp-52k	BAL22342	[13]
				Mrcp-100k	BAB12269	[14]
			*Balanus albicostatus*	Balcp-19k	AB242295	[11]
				Balcp-20k	AB329666	[15]
			*Balanus improvisus*	Bicp-19k	AB242296	[11]
spider		Araneae	*Nephila clavipes*	ASG1	EU780014	[16]
				ASG2	EU780015	[16]
				PySp2	HM020705	[17]
			*Latrodectus hesperus*	AgSF1	JX262195	[18]
				AgSF2	JX262192	[18]
				PySp1	FJ973621	[19]
tick		Acari	*Rhipicephalus appendiculatus*	RIM36	AY045761.1	[20]
				64P	AF469170	[21]
			*Rhipicephalus haemaphysaloides*	RH50	AY550980	[22]
mussel	Mollusca	Bivalvia	*Dreissena polymorpha*	Dpfp1	AAF75279	[23]
				Dpfp2	AM229730	[24]
			*Mytilus californianus*	Mfp-3S	DQ165556	[25]
				Mcfp-5	DQ444853	[26]
				Mcfp-6	DQ351537	[26]
			*Mytilus edulis*	Mefp-1	AY845258	[27]
				Mefp-3	AF286136	[28]
				Mefp-5	AAL35297	[29]
			*Mytilus galloprovincialis*	Mgfp1	D63778	[30]
				Mgfp5	AY521220	[31]
			*Perna viridis*	Pvfp-1	AAY46226	[32]
				Pvfp-2	AGZ84282	[7]
				Pvfp-3	AGZ84285	[7]
				Pvfp-5	AGZ84279	[7]
				Pvfp-6	AGZ84283	[7]
slug		Gastropoda	*Lehmannia valentiana*	Sm40	ABR68007	[33]
				Sm85	ABR68008	[33]
tubeworm	Annelida	Polychaeta	*Phragmatopoma californica*	Pc-1	AAY29115	[34]
				Pc-2	AAY29116	[34]
				Pc-3A	AY960618	[34]
				Pc-3B	AY960621	[34]
				Pc-4	GH160602	[35]
				Pc-5	GH160603	[35]
			*Sabellaria alveolata*	Sa-1	CCD57439	[36]
				Sa-2	CCD57460	[36]
				Sa-3A	CCD57480	[36]
				Sa-3B	CCD57502	[36]

#### 2.1.1. Echinoderms

Echinoderms are among the most familiar marine creatures, and representatives, such as the sea stars (figure 1*a*), have become virtually a symbol of sea life. In sea stars and sea urchins, adhesion takes place at the level of a multitude of small appendages, the tube feet (or podia), and involves the secretion of an adhesive between these specialized organs and the substratum [37]. The tube feet are the external appendages of the ambulacral system of these organisms and are used in locomotion and attachment. They consist of a proximal extensible stem and a distal disc-shaped extremity. The stem allows tube foot movements while the disc mediates adhesion to the substratum through the secretion of an adhesive material from one or two types of adhesive cells [38]. Adhesion is temporary, however, and after the tube foot has become voluntarily detached, the adhesive material remains firmly bound to the substratum as a footprint [39,40]. At present, data on the biochemical composition of echinoderm adhesive are only available for the sea star *Asterias rubens* and the sea urchin *Paracentrotus lividus* [39,41]. The water content of the adhesive material has never been measured but, in terms of dry weight, the adhesive material is mainly made up of proteins (20.6% in sea stars and 6.4% in sea urchins), carbohydrates (8% in sea stars, 1.2% in sea urchins) and a large inorganic fraction (approx. 40% in sea stars, 45.5% in sea urchins) [39,41]. In both species, potential novel adhesive proteins have been extracted [41,42]. However, only one has been completely sequenced: the protein Sfp1 from sea stars [8]. This protein, a primary constituent of the footprints, consists of four subunits, each displaying specific domains that mediate interactions with other proteins present in the adhesive material and on the tube foot surface. Sfp1 forms a structural scaffold and appears to provide cohesion to the adhesive layer [8].

#### 2.1.2. Barnacles

Barnacles (figure 1*b*) are marine sessile crustaceans that attach firmly and in large numbers to a variety of underwater natural substrata such as rocks, and also to man-made substrata such as ship hulls, leading to increased fuel consumption, requiring regular cleaning, and therefore causing major economic losses. In these organisms, attachment is mediated by the release of a permanent adhesive called cement [43,44]. This cement is produced by large isolated secretory cells (the cement cells) joined together by ducts which open onto the base of the animal [45–47]. The cement is composed of approximately 90% proteins with the remainder being carbohydrates (1%), lipids (1%) and inorganic material (4%, of which 30% is calcium) [48]. More than 10 proteins have been identified in the cement (cement proteins abbreviated as cp), of which six have been purified and characterized, originally from the species *Megabalanus rosa* and later from other species [44,49,50]. Among these proteins, three (cp-19k, cp-20k and cp-68k) have a surface coupling function, two (cp-52k and cp-100k) have a bulk function, and the last one (cp-16k) is an enzyme whose possible function is the protection of the cement from microbial degradation [44,49,50].

#### 2.1.3. Tubeworms

Some marine worms (figure 1*c*) of the family Sabellariidae are tube dwelling and live in the intertidal zone [51]. They are commonly called honeycomb worms or sandcastle worms because they are gregarious and the tubes of all individuals are closely imbricated to form large reef-like mounds. To build the tube in which they live, they collect with their tentacles particles such as sand grains or shell fragments from the water column and sea bottom. These particles are then conveyed to the building organ, which is a crescent-shaped structure near the mouth. There, the particles are dabbed with spots of cement secreted by two types of unicellular glands (cells with homogeneous granules and cells with heterogeneous granules), and they are added to the end of the pre-existing tube by the building organ [52–54]. Cement composition has been extensively investigated in the species *Phragmatopoma californica*, and consists mostly of several different proteins, a sulfated macromolecule and large amounts of Mg^2+^ and Ca^2+^ ions [34,35,54,55]. The five major cement proteins, named *Phragmatopoma* cement proteins (abbreviated as Pc-1 to Pc-5), have highly repetitive primary structures with limited amino acid diversity [34,55,56]. Pc-1, Pc-2, Pc-4 and Pc-5 are all basic. Pc-3 is characterized by the overabundance of serine residues which are largely phosphorylated. Pc-3 is therefore extremely acidic. Tyrosine residues of both Pc-1 and Pc-2 are post-translationally modified into 3,4-dihydroxyphenylalanine (DOPA) [57]. DOPA groups take part in surface coupling either through hydrogen bonds or by forming complexes with metal ions and metal oxides present in mineral surfaces [58]. Following oxidation, DOPA groups also contribute to cement curing by forming intermolecular cross-links [34]. The different cement components are packaged and stored in concentrated granules in the two cell types. Homogeneous granules contain the sulfated macromolecules and the proteins Pc-2 and Pc-5, whereas heterogeneous granules contain the proteins Pc-1, Pc-3 and Pc-4, paired with divalent cations. Co-secretion and limited mixing of the preassembled adhesive packets lead to formation of a complex composite cement in which the localization and role of the different adhesive proteins are still poorly understood [59].

#### 2.1.4. Mussels

Mussel is the common name used for members of several families of bivalve molluscs, from both marine and freshwater habitats. To attach themselves to the substratum, mussels produce a byssus (figure 1*d*), which consists of a bundle of proteinaceous threads, each connected proximally to the base of the animal's foot, within the shell, and terminating distally with a flattened plaque which mediates adhesion to the substratum [3,27]. The composition of these plaques has been mostly characterized from marine mussels of the genera *Mytilus* and *Perna*. They are formed by the auto-assembly of secretory products originating from four distinct glands enclosed in the mussel foot. These products comprise a collagenous substance, a mucous material, a mixture of polyphenolic proteins (known as foot proteins 2 to 6, abbreviated as fp-2 to fp-6) and an accessory protein (fp-1) [3,7,26,60–62]. Since the characterization of fp-1 in the early 1980s, *Mytilus* foot proteins have been the subject of a very large number of studies leading to a detailed knowledge on their structures, functions and interactions within the byssal attachment plaque. Proteins fp-2 and fp-4 form the central core of the plaque; fp-3, fp-5 and fp-6 are located at the interface between the plaque and the substratum (primer layer); and fp-1 forms a hard cuticle protecting the core from hydrolysis, abrasion and microbial attack [27,58,62,63]. Among the latter, the presence of DOPA is a common distinctive feature shared by all the proteins identified in the byssal plaque. This modified amino acid fulfils the same roles as in tubeworm adhesive proteins: it is involved in the formation of cross-links between the different fps (cohesion) and it mediates physico-chemical interactions with the surface (adhesion) [58].

Among freshwater mussels, adhesion has been investigated mostly in zebra mussel (*Dreissena polymorpha*) because it is a highly invasive species. Although the zebra mussel byssus is superficially similar to the one of marine mussels, significant structural and compositional differences have been reported [64]. For example, contrary to the situation in marine mussels, the composition of the zebra mussel thread and plaque appears to be quite similar. To date, 13 zebra mussel proteins (Dpfp-0 to Dpfp-12) have been characterized that do display similarities to the marine fps including the presence of DOPA [23,24,65,66].

#### 2.1.5. Sticklebacks

Three-spined sticklebacks, *Gasterosteus aculeatus* (figure 1*e*), are small freshwater fish that are used increasingly as models in evolutionary biology and ecology [67]. One fascinating aspect of stickleback biology is that the males build nests, which serve as receptacles for eggs and a focus for courtship. The male constructs his nest from plant materials, which are stuck together and to the substratum using an endogenous adhesive protein, spiggin, produced in the fish's kidney [9,68]. This protein is stored in the urinary bladder and, upon release, forms highly elastic adhesive threads. Spiggin is a cysteine-rich glycoprotein presenting a modular organization [9,68]. It is encoded by several distinct genes, some of which presenting splicing variants [69]. It is proposed that the presence of a multi-gene family coding for spiggin could contribute to the production of this protein in enough amounts to build nests during the breeding season [69]. Recently, Seear *et al.* [70] showed that spiggin genes expression is significantly affected by both the flow regime experienced by the fish and its nesting status.

#### 2.1.6. Slugs

Slugs are terrestrial gastropod molluscs (figure 1*f*) which attach to surfaces by secreting a viscous mucus film on which they creep by mean of waves of muscular contractions running along their foot [71,72]. The so-called trail mucus is left behind by the slug as it moves. In *Lehmannia valentiana*, this adhesive mucus is principally composed of water (approx. 90%) while the remaining 10% consists mainly of carbohydrates (34–41% of dry weight) and proteins (25–34% of dry weight) [33]. Among the 18 proteins making up the protein fraction, two have been sequenced, Sm40 and Sm85. Both contain specific domains which could promote protein–protein interactions [33].

#### 2.1.7. Velvet worms

Velvet worms are terrestrial soft-bodied invertebrates inhabiting tropical and temperate forests. To defend themselves against predators or to capture their prey, these organisms use a sticky secretion produced by large glands located on each side of the gut within the body cavity and expelled via a pair of modified limbs called slime papillae (figure 1*g*) [73,74]. This secretion is ejected as adhesive threads forming a net entangling the prey [75,76]. To date, biochemical analyses have focused on the composition of the slime produced by velvet worms from the genus *Euperipatoides*. This slime is principally composed of water (approx. 90%) while the remaining 10% consists mainly of proteins (55% of dry weight) associated to carbohydrates (1.3%), and small quantities of lipids and the surfactant nonylphenol [75]. In the species *Euperipatoides rowelli*, the protein fraction is mainly made up of high molecular weight proline-rich proteins (Er_P1, Er_P2 and Er_P3). Carbohydrate-binding proteins and small peptides are also present in the adhesive secretion where they could act as antimicrobial agents and protease inhibitors [10].

#### 2.1.8. Spiders

Spiders (figure 1*h*) are able to spin high performance silk fibres that they use for a wide range of functions, including prey capture, locomotion and protection of eggs. In Araneomorphae, seven distinct glands comprise the spinning apparatus. Among them, two are involved in adhesion: the pyriform gland produces attachment disc silks, which attach dragline silk to substrates, and the aggregate gland produces an aqueous glue which covers the silk fibres of the web as well as an adhesive which lash the connection joints of the web [16,18,19]. The pyriform secretion from orb web and cobweb spiders is a gelatinous substance, which dries to form a strong chemical adhesive [19]. It is composed of adhesive proteins (pyriform spidroins 1 and 2) that contain different internal block repeats (including proline-rich segments) and share a high percentage of polar amino acids within their protein sequences [17,19,77]. The viscid glue produced by the aggregate glands of orb-weaving spiders functions primarily to retain flying insects. It is a complex assembly of glycoproteins and an aqueous solution of low molecular weight hygroscopic salts that regulate water content in the drop and keep the glycoproteins soft and tacky to maintain the stickiness in variable humidity environments [78,79]. The glycoprotein component is composed of two unique protein subunits (aggregate spider glue 1 and 2) that are both glycosylated. ASG1 has a high proportion of charged amino acids and is highly similar to chitin-binding proteins, while ASG2 has similarities with elastin and is thus associated with elasticity [16]. In the cobweb spider *Latrodectus hesperus*, the aggregate gland also produces two proteins, AgSF1 and AgSF2, that interface with dragline silks to form the connection joints of the web [18]. Both proteins are non-glycosylated and present internal amino acid block repeats.

#### 2.1.9. Ticks

Ticks are small, ectoparasitic acarids (figure 1*i*), living by haematophagy on the blood of mammals and birds. To feed on their hosts, Ixodid ticks rely on the secretion of a cement to remain attached on the host skin for the complete duration of their blood meal. The formed cement cone extends from the tick hypostome to the host epidermis. It is primarily proteinaceous, and also contains some carbohydrate and lipid [20,80]. The protein fraction appears to be made up of proteins rich in glycine, the number of which varies according to the species [20,80–82]. Some of these proteins, generally defined as glycine-rich proteins, have been characterized and tested as anti-tick vaccines [20,80,81]. In *Rhipicephalus appendiculatus*, two cement proteins, RIM36 and 64P, have been characterized. They possess a high content of glycine, serine and proline, and contain two types of glycine-rich amino acid repeats [20,21].

### 2.2. Biosynthesis

**Figure 2.**
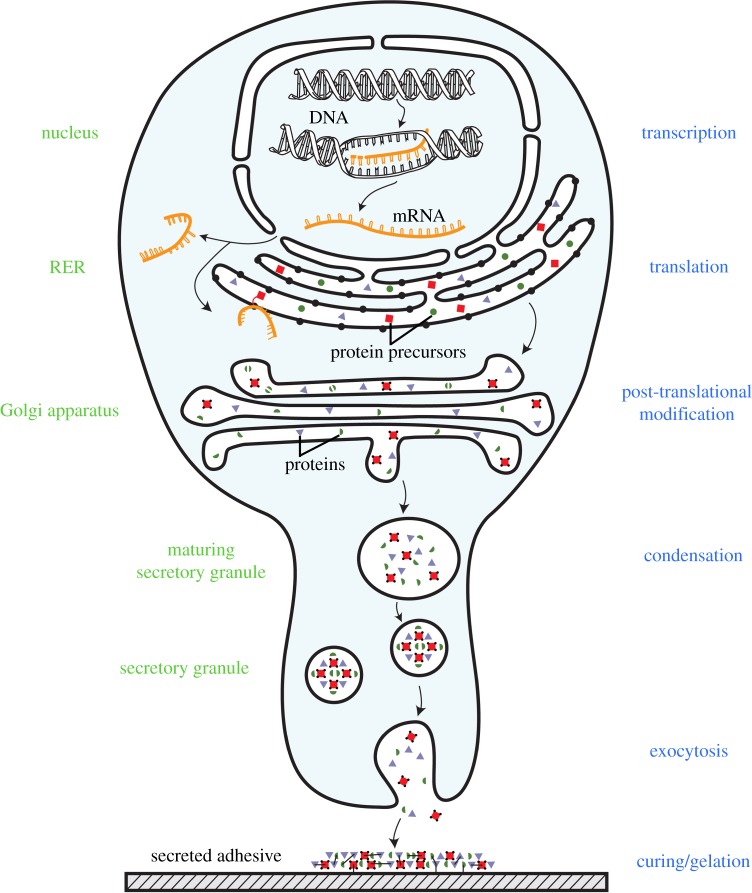
Schematic illustration of the synthesis and secretory pathway followed by adhesive proteins in a typical adhesive cell (not to scale). The names of the different molecules, cellular compartments and processes involved are indicated in black, green and blue, respectively. See text for a detailed description.

The biosynthesis, packaging and release of the adhesive proteins by the adhesive organs follow the so-called regulated secretory pathway [83]. This pathway is summarized in figure 2 for a typical adhesive cell. In the nucleus, the genes encoding adhesive proteins are transcribed into mRNAs, which are then matured and exported to the cytosol where they are translated at the level of ribosomes. The rough endoplasmic reticulum (RER) then captures secreted proteins from the cytosol as they are being synthesized. Indeed, these proteins possess a polypeptide signal sequence that directs the engaged ribosome to the endoplasmic reticulum membrane. The newly formed protein is then fully translocated across the RER membrane and released into its lumen. All adhesive protein precursors described so far present such a signal peptide, which is cleaved off in the RER. Proteins are then transferred from this compartment to the Golgi apparatus and from the Golgi apparatus to immature secretory granules by means of transport vesicles. As the granules mature, their contents become concentrated, probably as the result of both the continuous retrieval of membrane and the progressive acidification of the granule lumen. In adhesive cells, it has been suggested that protein condensation could involve a process called complex coacervation which is the spontaneous fluid–fluid phase separation of an aqueous protein solution into two immiscible aqueous phases, a dilute equilibrium phase and a denser, protein-rich coacervate phase [84]. In sandcastle worms, given the presence of both polyanions (acidic proteins) and polycations (basic proteins) in the granules, coacervation is driven by the electrostatic attraction and neutralization of these oppositely charged polyelectrolytes [34,55]. In mussels, polyanions are not known to be involved in adhesion but it has been demonstrated that a zwitterionic variant of the protein fp-3 (Mfp-3S [85]) can coacervate with itself through both electrostatic and hydrophobic interactions [86]. Because their final mature secretory granules are so densely filled with contents, adhesive cells can release large amount of material promptly by exocytosis when triggered to do so.

During their transfer from one compartment to the next, proteins are successively modified. Post-translational modification (PTM) is indeed a common feature of many adhesive proteins (see §3.3). One such modification, *N*-glycosylation, takes place in the endoplasmic reticulum, with subsequent oligosaccharide processing in the Golgi apparatus [83]. Many other PTMs, such as *O*-glycosylation or phosphorylation, occur in the Golgi apparatus [83]. Another possible PTM is protein cleavage. In sea stars, cleavage of the adhesive protein Sfp-1 apparently is autocatalytic, occurring at low pH in the late secretory pathway (in the Golgi apparatus or in the maturing secretory granules) of the tube foot adhesive cells, and generating four protein subunits [8].

Upon release, the adhesive proteins spread readily on the substrate where they auto-assemble to form the adhesive joint. This formation is usually accompanied by a gelation or curing process of the adhesive. For terrestrial organisms in which the adhesive is released in the form of a liquid protein solution, gelation could be triggered by water loss owing to evaporation. This model has been proposed to explain the formation of velvet worm slime, in which water evaporation would bring hydrophobic domains and regions of opposite charge from the proline-rich adhesive proteins in closer proximity to enable ionic and hydrophobic interactions [10]. In aquatic organisms, gelation could result from a pH or ionic strength differential between the secretory granules and water. Many marine adhesive proteins are post-translationally modified with different chemical groups. Among those, phosphates and sulfates may be involved in non-covalent adhesive and/or cohesive interactions, possibly through Ca^2+^ or Mg^2+^ bridging. In the cement of the tubeworm *P. californica*, which contains the polyphosphorylated protein Pc-3 [34], the jump in pH accompanying secretion, from about 5 in the secretory granule to 8.2 in seawater, could trigger a change in bonding between Ca^2+^ and phosphate from electrostatic to ionic, the effect of which would be to harden spontaneously and solidify the adhesive [87].

Creating covalent cross-links between constitutive macromolecules is another way to cure the adhesive secretion. In DOPA-containing adhesives, such as the tubeworm cement or the mussel byssal plaque, cross-linking reactions follow the oxidation of DOPA to DOPA-quinone, a reaction catalysed by a polyphenol oxidase (catechol oxidase^1^). Once formed, DOPA-quinone is capable of participating in a number of different reaction pathways leading to intermolecular cross-link formation [7,34,88]. Secreted catechol oxidases have been detected in both the tubeworm cement and the mussel byssus [55,89]. In barnacles, Dickinson *et al.* [90] proposed that cement polymerization could depend on glutamyl-lysine cross-linking mediated by a transglutaminase but this hypothesis was later refuted by Kamino [50].

## 3. Molecular tools to characterize protein-based adhesives

**Figure 3.**
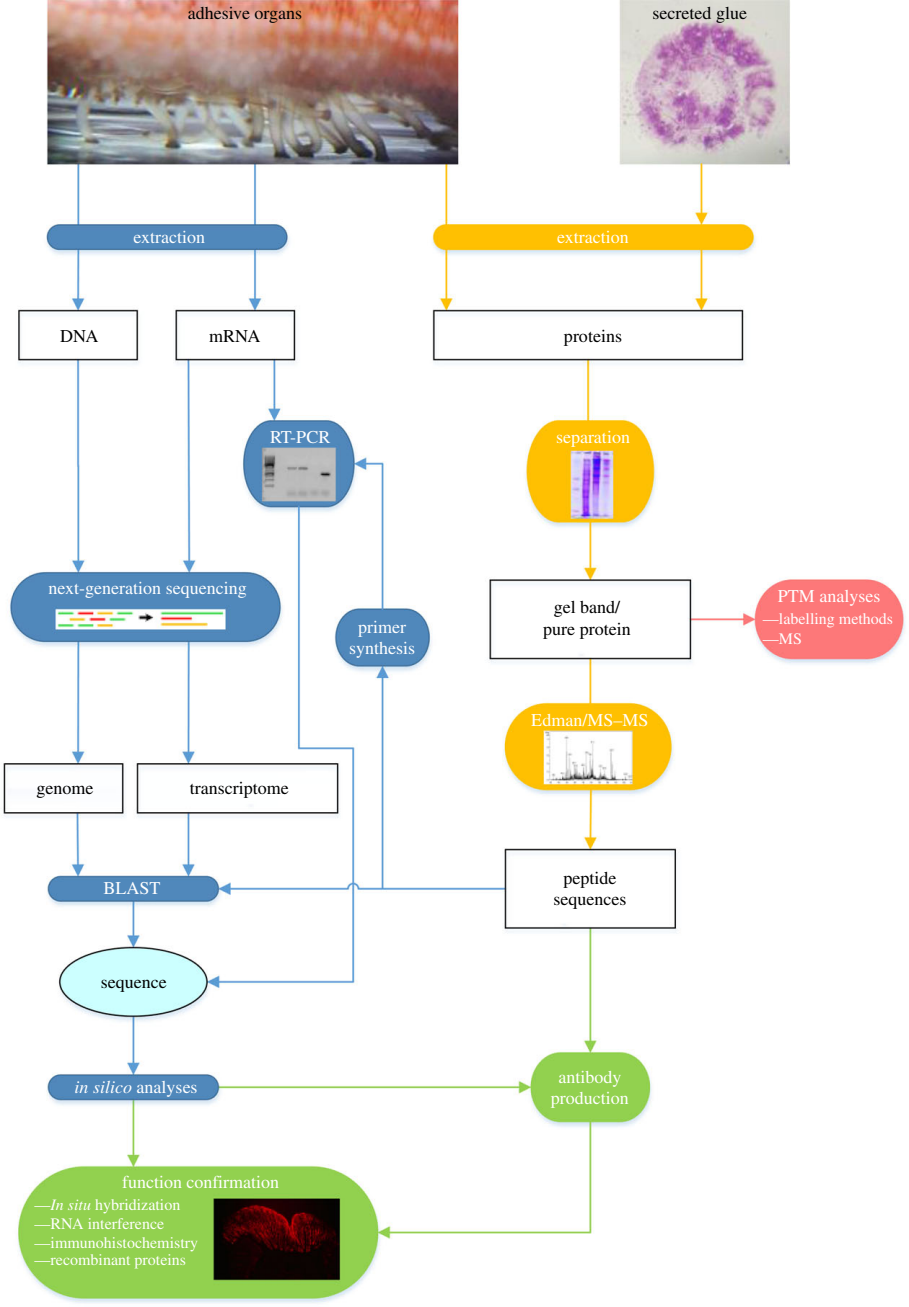
Molecular tools used to characterize protein-based adhesives, illustrated by examples from sea stars. Nucleic acids (DNA and mRNA; blue pathway) are extracted from the adhesive organ(s) (here, tube feet). Both can be submitted to next-generation sequencing to obtain, respectively, the genome of the animal or the transcriptome of the adhesive organ(s). Proteins (yellow pathway) are extracted from the secreted material (here, adhesive footprints) or the adhesive organ(s). After purification, they can be analysed for the presence of post-translational modifications (PTMs; red pathway) and/or submitted to peptide sequencing by Edman degradation or tandem mass spectrometry (MS/MS). The peptide sequences can be directly used for a BLAST search in the genome or transcriptome, or to design degenerate primers to perform RT-PCR, both allowing the recovery of the sequence of the cDNA coding for the investigated protein. The sequence is then analysed *in silico* to extract information required to perform experiments allowing the validation of the adhesive function of the protein (green pathway).

On the basis of the adhesive cell secretory pathway (figure 2), two different strategies can be used to characterize putative adhesive proteins: molecular biology tools allow the retrieval of complete primary sequences from the cell nucleotidic information (DNA or mRNA) while protein chemistry tools permit one to obtain polypeptidic sequences and PTM information directly from the secreted proteins or from their precursors in the cells (figure 3). Although these two strategies can be used independently (e.g. protein chemistry approach for the mussel protein Mefp-3 [91]; molecular biology approach for the tubeworm proteins Sa-1 to Sa-3 [36]), they are generally conducted in parallel because the combination of their respective results facilitates the identification and characterization of the adhesive protein candidates.

### 3.1. Identification of adhesion-related genes and mRNAs

To the best of our knowledge, no adhesive protein has ever been identified directly from an animal's genome. The identification and isolation of adhesion-related genes can indeed be hampered by the lack of information or sequence data for the respective organism. On the other hand, for many organisms, there is no genomic database available. Therefore, mRNA sequencing is an essential prerequisite to get access to the genes expressed in certain tissue. Until recently, this was achieved by constructing a complementary DNA (cDNA) library and sequencing clones randomly to generate expressed sequence tags (ESTs; e.g. [92]). However, with the advent of next-generation sequencing (NGS), access to mRNA information is now obtained directly by transcriptome sequencing (e.g. [93,94]).

A cDNA library is a combination of cloned cDNA fragments inserted into a collection of host cells (generally bacteria), which together constitute some portion of the transcriptome of the organism or the tissue investigated. The generation of cDNA libraries involves the following steps: (i) isolation of mRNA, (ii) reverse transcription of mRNA into cDNA, and (iii) ligation of cDNA fragments into bacterial plasmids. Stewart and co-worker [35] took advantage of a cDNA library constructed from the adhesive gland of the tubeworm *P. californica* to identify potential novel adhesive proteins. As mRNAs are present in high number of copies for proteins produced continuously by the adhesive cells, they hypothesized that cDNAs coding for adhesive proteins should be more represented in the library than other cDNAs. They sequenced about 300 random clones and analysed the sequences using bioinformatics tools (see below). This approach allowed the identification of 18 genes encoding proteins with characteristics of secreted structural proteins, including the most abundant cement proteins Pc-1 to Pc-5 [35,56]. Some specific knowledge about the composition of the adhesive secretion can also be useful to obtain the full-length sequence of particular adhesive proteins. This is exemplified by the work of Zhao *et al.* [34], who compared the amino acid analysis of whole tubeworm cement to the amino acid composition of its two constitutive proteins known at that time (Pc-1 and Pc-2), and, by doing this, deduced that a third protein containing more than 80 mol% of serine was probably present in the cement. Therefore, they designed a degenerate primer corresponding to five consecutive serine residues (using the codon preference for serine in Pc-1 and Pc-2), and used it to amplify by PCR the sequence of the protein Pc-3 from a cDNA library of the tubeworm cement gland. Choresh *et al.* [16] used similar methods (random sequencing and PCR) to screen the aggregate gland cDNA library from the spider *Nephila clavipes*. They identified an abundant clone coding for two proteins: ASG1 and ASG2. The selection of clones representing highly expressed proteins can also be done through DNA microarray analysis (e.g. identification of the proteins AgSF1 and 2 from the aggregate gland of the spider *L. hesperus* [18]). In ticks, a 15 kDa cement protein, 64P, was identified in a cDNA library prepared from the salivary glands of adult female *R. appendiculatus*, based on its amino acid composition [21,95].

**Figure 4.**
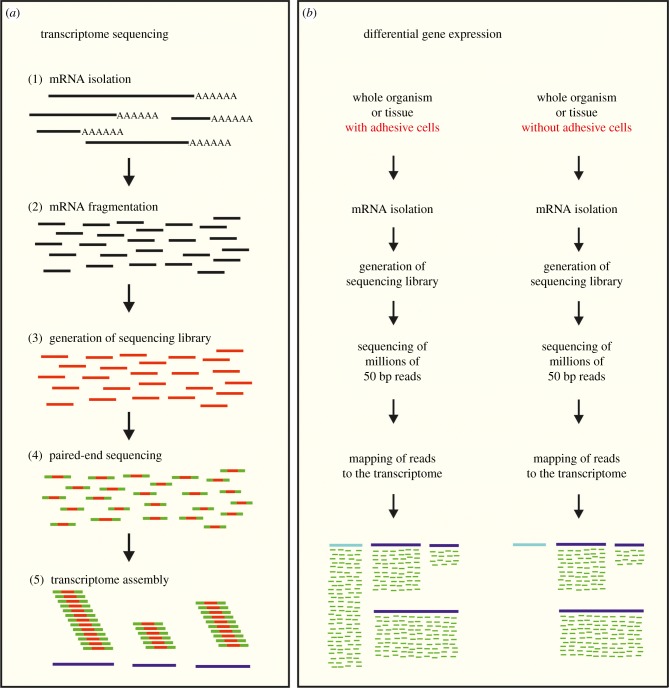
Consecutive steps for transcriptome sequencing (*a*) and experimental set-up for differential gene expression (*b*). See text for details. (*a*) mRNA (black); sequencing library (red), note adapters at the ends are not shown; paired-end reads (green–red–green), each sequenced stretch at both ends (green) of the DNA fragment (red) is about 100 bp assembled transcripts (purple lines). (*b*) Transcripts of transcriptome (purple lines) and adhesive transcript (blue line). Note that no reads can be mapped to the adhesive transcript when tissue without adhesive cells used.

Generation of a transcriptome using a short reads NGS method involves several steps (figure 4*a*): (i) isolation of mRNA, (ii) fragmentation of mRNA, (iii) generation of a sequencing library with adapters on both ends, (iv) paired-end sequencing, and (v) bioinformatic assembly of paired-end reads. While a transcriptome provides the full complement of expressed transcripts, an adhesion-related tissue- or cell-type specific candidate gene list would be favourable. For this purpose a differential RNA-seq approach [96–99] can be applied (figure 4*a*), especially if only small amounts of adhesive tissue can be collected. Briefly, tissue containing adhesive cells and tissue lacking adhesive cells are sequenced using short reads (50 bp). These reads are mapped to the existing transcriptome to identify differentially expressed transcripts, both on a qualitative and on a quantitative level [100]. This differential approach allows one to identify candidate adhesive genes in the absence of protein information (molecular weight, composition or sequence), and is therefore of particular interest when the amount and/or insolubility of the adhesive material hinders protein extraction (see also below). In the marine flatworm *Macrostomum liguano*, the differential RNA-seq approach allowed the identification of an intermediate-filament gene which, although it does not code for an adhesive protein, is essential for the adhesive process [101]. In barnacles, high-throughput NGS has been applied to compare transcriptomes of the prosoma (non-adhesive tissue) and the basis (which contains the cement gland) of the membranous-based species *Tretraclita japonica formosana* [102]. The authors showed that homologues of barnacle cement proteins cp-19K, cp-52K and cp-100K as well as adhesion-related genes such as crustin or fibroin were predominately expressed in the basis. Likewise, existing adhesive protein sequences available in publicly accessible databases can be very valuable for the identification of adhesion-related genes in related species through bioinformatic comparisons. For instance, Guerette *et al.* [7] sequenced the transcriptome of the foot of the green mussel *Perna viridis*, and conducted BLAST homology searches of all known *Mytilus* foot proteins against it. By doing so, they retrieved the full-length sequences of two adhesive proteins from *P. viridis*: Pvfp-1 and Pvfp-2. Other protein characteristics such as molecular weight or composition can also be used to help retrieve sequences from transcriptomes (e.g. Pvfp-3, Pvfp-5 and Pvfp-6 [7]).

When the sequence of an adhesive protein has already been characterized in a closely related species and high protein homology is likely, straightforward techniques of molecular biology such as RT-PCR can be used to obtain the full-length sequence of putative adhesive proteins without the need to sequence a high number of transcripts (figure 3). For example, Becker and co-workers [36] designed primers from the known cDNA sequences coding for the proteins Pc-1, Pc-2 and Pc-3A and Pc-3B of the tubeworm *P. californica* to target the cDNA coding for the homologous proteins Sa-1, Sa-2 and Sa-3A and Sa-3B in *Sabellaria alveolata*. In barnacles, a similar approach was used to identify the protein homologous to Mrcp-19k from *M. rosa* in *B. albicostatus* (Balcp-19k) and *B. improvisus* (Bicp-19k) [11]. Advantage can also be taken of the presence of repeated amino acid sequences in the known adhesive proteins. Degenerate primers can be designed on the basis of these sequences and be used in PCR in order to identify the cDNA sequence of homologous proteins in close species (e.g. the protein from *Mytilus galloprovincialis* Mgfp1 homologous to *Mytilus edulis* fp-1 [30]; and the protein from *B. albicostatus* Balcp-20k homologous to *M. rosa* cp-20k [15]). These different strategies require however that the adhesive proteins from the different species present sufficient sequence identity between each other.

Once the complete primary sequence of a putative adhesive protein has been obtained, its characterization usually starts by *in silico* analyses. A large number of bioinformatics tools are available for making predictions about the function and physico-chemical properties of a protein on the basis of its primary sequence (see, for instance, the ExPasy proteomics server [103]). These tools are of particular interest in the field of bioadhesion, where sequence characteristics are generally recognized as major contributors to protein adhesive properties. One major tool used in most studies is SignalP [104], which predicts the presence of the signal peptide, a short (5–30 amino acid long) hydrophobic sequence present at the N-terminal part of proteins destined to be secreted (see §2.2). Identification of the so-called secretome is an approach that has been used in tubeworms, for example [35]. The analysis of amino acid composition of a protein (e.g. with ProtParam [105]) allows one to reveal the presence of highly expressed amino acids (e.g. the glycine- or serine-rich proteins in the tubeworm *P. californica* [35]; or the glycine-rich proteins of ticks [80]) and the resulting isoelectric point (pI) of the protein (e.g. the basic proteins Sa-1 and Sa-2 in the tubeworm *S. alveolata* [36]). The presence of repeated amino acid sequences is a frequent feature in adhesive proteins that can be identified using the Rapid Automatic Detection and Alignment of Repeats tool (RADAR [106]). For instance, this analysis has been used to identify the tandem repeats made up of consecutive charged residues interspersed with short hydrophobic regions in the velvet worm proteins Er_P1 and Er_P3 [10]. In some species, adhesive properties appear to result from the presence of conserved structural domains able to link to other molecules, and which can be identified using, for example, the Conserved Domain Database from NCBI [107]. Indeed, many adhesive proteins display specific protein-, carbohydrate- and metal-binding domains: e.g. von Willebrand factor type D domain in the stickleback protein spiggin [68,69]; EGF-like and von Willebrand factor type A domains in the slug proteins Sm40 and Sm85 [33]; discoidin, von Willebrand factor type D, galactose binding, C8 and EGF-like domains in the sea star protein Sfp1 [8]. Finally, a number of tools are also available to predict potential sites of PTMs such as glycosylation, hydroxylation and phosphorylation in the primary sequence. Examples of the use of these tools can be found in Kawahara & Nishida [9] for the fish protein spiggin, and in Haritos and co-workers [10] for velvet worm adhesive proteins.

### 3.2. Identification of adhesive proteins

To date, most studies on adhesive proteins have been carried out on proteins isolated from two enriched sources: the secreted adhesive material or the adhesive gland/organ (figure 3). Although in some cases proteins can be easily isolated from the adhesive material (e.g. in velvet worms [75]), protein extraction is generally hindered by the insolubility of the adhesive material. Strong denaturing (e.g. urea, guanidine hydrochloride) and/or reducing (e.g. β-mercaptoethanol, dithiotreitol) conditions were indeed required to isolate proteins from spider webs [18], stickleback nest threads [69], mussel byssal adhesive plaques [108], barnacle cement [14] and sea star and sea urchin adhesive footprints [41,42]. To bypass the challenge of solubilizing the secreted cured adhesive material, it is sometimes more convenient to perform protein extraction on the dissected adhesive gland or organ, which are a source of soluble adhesive precursors (e.g. the stickleback kidney [68]; the mussel foot [26] or the sea urchin tube feet [109]). Once in solution, adhesive proteins must be purified, i.e. separated from non-protein parts of the mixture and from all other proteins. Purification steps exploit differences in protein size (e.g. separation by Sodium dodecylsulfate polyacrylamide gel electrophoresis [33]), size and isoelectric point (e.g. separation by two-dimensional polyacrylamide gel electrophoresis [110]), physico-chemical properties (e.g. precipitation with ammonium sulfate [108] or acetone [111]) and binding affinity (separation using different types of chromatography [57]).

Usually, the next step in adhesive protein identification is obtaining peptide sequences. Although several techniques exist to obtain partial amino acid sequence of a protein, the two dominant methods are Edman degradation and mass spectrometry (MS). Both methods have been successfully used to obtain the sequence of peptide fragments from adhesive proteins. These peptides result from the digestion of the purified protein (either in solution or in a gel band/spot) using chemical agents (e.g. cyanogen bromide [14]) or endopeptidases (e.g. trypsin or pepsin [41,112]). Peptides are usually desalted and/or separated by electrophoresis and/or chromatography before sequencing [109]. The Edman degradation method involves the chemical modification of the N-terminal amino acid of the peptide, which can then be cleaved from the chain and identified. Successive cycles of degradation are then used to identify the sequence of the peptide (e.g. mussel fps [108,111]). The same method can also be exploited to determine the N-terminal amino acid sequence of pure proteins (e.g. the tubeworm protein Pc-2 [57]) or of proteins separated by electrophoresis, transferred onto poly(vinylidene fluoride) or nitrocellulose membranes, and excised (e.g. the barnacle protein Mrcp-20k [12]).

In MS, biomolecules are ionized and their mass is measured by following their specific trajectories in a vacuum system [113]. Two fundamental strategies for protein identification and characterization by mass spectrometry are currently employed: top-down proteomics, in which intact proteins or large protein fragments are subjected to gas-phase fragmentation for MS, and a bottom-up proteomics, in which purified proteins, or complex protein mixtures, are subjected first to proteolytic cleavage, and what is analysed by MS are the peptide products (see [114–117] for review). Although MS can measure the mass of intact adhesive proteins (e.g. in mussel adhesive footprints [7,25]), it is usually used to sequence peptides resulting from enzymatically digested adhesive proteins. The two most popular techniques to identify protein sequences using MS are peptide mass fingerprinting (PMF) and tandem mass spectrometry (tandem MS or MS/MS). In the first method, the mass spectrometer generates a list of peptide masses which are compared with calculated peptide masses generated by *in silico* cleavage of protein or cDNA sequences present in publicly available databases using the same specificity as the enzyme that was employed experimentally (e.g. NCBI [118]). PMF can be performed with the same instrumentation used for MS/MS, but it is usually done using time-of-flight mass spectrometers with matrix-assisted laser desorption ionization. This method is however seldom used to identify adhesive proteins, because it requires pure proteins or simple mixtures of proteins, and several peptides are needed to unambiguously identify a protein. Tandem MS allows *de novo* internal sequencing, in which isolated peptides are fragmented and the generated mass spectra of resulting fragments are analysed to reconstitute the peptide sequences (e.g. slug proteins [33]; sea urchin and sea star proteins [41,42,109]; zebra mussel proteins [24,66]). Unique peptide sequences or all MS/MS data can be used for homology search in publicly available and/or home-made databases (e.g. transcriptomes and EST libraries (see §3.1), virtually translated into the six reading frames). A common problem is that the obtained MS or MS/MS spectra are typically matched against publicly available protein databases that in the case of most adhesive-producing organisms are nonexistent or derive from incomplete genome assemblies and annotations leading to deficient protein identification. For instance, protein isoforms arising from genetic polymorphisms, post-transcriptional events such as RNA-editing and PTMs will be largely missed (see [119] for review). This can be circumvented by coupling MS approaches with whole genome and total RNA sequencing, allowing the generation of near-complete databases of genetic variation and its transcripts for each adhesive organ (see §3.1). A dual proteomic and transcriptomic approach is therefore the best way leading to the identification of novel adhesive proteins and the retrieval of their complete sequences (e.g. the velvet worm protein Er_P1–3 [10]; the sea star protein Sfp1 [8]).

When the sequence of the unknown protein cannot be obtained from databases, a PCR approach can be employed. This technique relies on the design of degenerate oligonucleotide primers on the basis of the peptide sequences (figure 3). These primers are used to screen cDNA libraries (e.g. the mussel protein Mefp-5 [29]) or on mRNA reverse-transcribed into cDNA (RT-PCR) (e.g. the barnacle protein cp52k [13]). In this strategy, primers designed on N-terminal peptides are more convenient as only sense degenerate primers are used in combination with antisense universal primers encoded in the vector used to build the cDNA library (e.g. Mefp-5 [29]) or encoded by a specific adapter incorporated during the RT (e.g. the tubeworm proteins Pc-1 and Pc-2 [34]; the slug proteins Sm40 and Sm85 [33]). Degenerate primers designed on internal peptides, however, have to be tested as sense or antisense. RT-PCR can also be performed using a sense degenerate primer designed on an N-terminal peptide and an antisense degenerate primer designed on an internal peptide (e.g. the zebra mussel protein Dpfp1 [23]), or using two primers designed on both extremity of a same peptide (e.g. the barnacle protein Mrcp-100k [14]). Discrete PCR products obtained following these strategies are subsequently cloned and sequenced. The generated partial sequences are usually completed by 3′- and 5′- rapid amplification of cDNA ends (RACE)-PCR using sequence specific primers (e.g. the barnacle protein Mrcp-19k [11]). The full-length sequence of the adhesive protein is then deduced from the open reading frame of the cDNA sequence which, in eukaryotes, starts with an ATG codon (coding for methionine) and ends with a stop codon.

### 3.3. Identification of post-translational modifications

The physico-chemical properties of many adhesive proteins are derived in part from their PTMs [87,120]. In the adhesive proteins described so far, these modifications are of three main types: glycosylation, hydroxylation and phosphorylation.

#### 3.3.1. Glycosylation

An impressive variety of carbohydrate–peptide linkages have been described from glycoproteins found in essentially all living organisms. These glycopeptide bonds can be classified in different groups, the most common being the *N*-glycosidic bonds in which the oligosaccharide is attached to the amide group of an asparagine residue within the consensus peptide sequence NXS/T, where X is any amino acid except proline; *O*-glycosidic bonds in which a mono- or oligosaccharide is attached to the hydroxyl group of a serine or threonine residue; and C-mannosyl bonds in which a mannose residue is attached to a tryptophan residue through a C–C bond (see [121,122] for review). Glycoproteins have been implicated in attachment processes of fouling organisms such as barnacles and mussels. Examples include the freshwater mussel adhesive proteins Dpfp-1 and Dpfp-2 [65], which present extensive threonine or serine *O*-glycosylation. The marine mussel adhesive protein Pvfp-1 was shown to possess not only extensive threonine *O*-glycosylation but also tryptophan mannosylation [32,123]. The roles of the sugar residues in mussel proteins are still speculative, but have been proposed to increase conformational stability and enhancement of protein binding ability [122]. C-linked mannosylation would indeed render tryptophan more polar and solvent-accessible. It was also observed to block cleavage in the vicinity of modified residues by exo- and endoproteases, hence rendering proteins more resistant to degradation [32]. In barnacles, one cement protein, cp-52k, is known to be *N*-glycosylated [13].

The analysis of protein glycans is complicated by their vast variety and the large number of potential glycosylation combinations. Moreover, the same glycosylation site can be occupied by different glycans in different copies of a protein (see [122] for review). Despite the detection of glycoproteins in the adhesives of a wide range of organisms, little is still known about the composition of their carbohydrate fraction and whether these carbohydrates are covalently attached to the proteins or not. Below are some of the methods that have been applied to bioadhesives.

*Detection by staining*. A basic, simple method to determine whether a protein is glycosylated is to resolve it by one-dimensional or two-dimensional electrophoresis and to stain the gel for glycoproteins. Most gel-staining procedures are based on the periodic acid–Schiff reaction, in which periodic acid oxidizes vicinal diols in carbohydrate residues to form two aldehyde groups, which react with the Schiff reagent to give a magenta colour. This reaction was used by Ohkawa *et al.* [123] to demonstrate the presence of *O*-glycosylated threonines in Pvfp-1. Other reactions use periodate oxidation followed by biotinylation and binding of peroxidase-labelled avidin. This method was used by Kamino *et al.* [13] to highlight the *N*-glycosylation of cp-52k. Affinity-based staining can also be performed using lectins which are proteins that specifically bind mono- or oligosaccharides, allowing glycoprotein detection and characterization through lectin histochemistry and lectin bloting. Hennebert *et al.* [124] used a set of 16 lectins to label tube foot sections, footprints, and proteins extracted from these footprints in the sea star *A. rubens*. One *N*-linked and one *O*-linked glycoprotein were detected, their oligosaccharide chains enclosing galactose, *N*-acetylgalactosamine, fucose and sialic acid residues.

*Glycan structure analysis*. Once protein glycosylation has been confirmed, the glycan moiety structure can be further studied by liquid chromatography and MS. The glycan can be analysed either attached to the protein or following its release, the latter being more reliable. Depending on its nature, the glycan moiety can be removed from the protein by chemical or enzymatic means. For chemical removal, the two main methods for removing *O*- and *N*-linked oligosaccharides are β-elimination and hydrazinolysis, respectively. Amino acid analysis of Dpfp-1 and Dpfp-2 following removal of carbohydrate residues by reductive β-elimination allowed Rzepecki & Waite [65] to detect heavy loss of threonine and serine residues, suggesting that approximately 76% of the *O*-glycans in Dpfp-1, and some 90% in Dpfp-2, were attached to threonine rather than serine. For enzymatic cleavage, most of the commercially available enzymes are specific for *N*-glycans, with fewer being available for *O*-glycans. In addition, given the diversity of *O*-glycans several enzymes might be required for the analysis of a single sample, and in most cases chemical removal is preferable. The most used *N*-glycan-cleaving enzymes are PNGase F and A, or endoglycosidases such as Endo-H. After its release, the glycan moiety can be analysed by chromatography using a fluorescent label to improve its detection. Glycan chromatography is often coupled with mass spectrometry for the elucidation of glycan sequence, branching and linkage [125]. MS can also be used to analyse the protein moiety from which the glycan was removed, in order to identify the amino acids that were formerly attached to the glycans. Tandem MS was used to demonstrate that, in mussel Pvfp-1, some tryptophan residues are C2-mannosylated [32].

#### 3.3.2. Hydroxylation

During the chemical process of hydroxylation, an amino acid residue is modified by the attachment of at least one hydroxyl group (–OH). Hydroxylation of amino acid side chains in proteins is less common than other PTMs such glycosylation or phosphorylation [126,127]. Protein hydroxylation is however an important process in marine bioadhesion. Up to now, four amino acids have been found to be hydroxylated in mussel and tubeworm adhesive proteins, giving rise to five modified residues. These residues seem to confer to marine proteins their exceptional adhesive properties. Indeed, they may contribute to make the protein more competitive with water by creating hydrogen bonds with surfaces [58,91,128]. DOPA is formed by the hydroxylation of tyrosine residues by a polyphenol oxidase (tyrosinase^1^). This modified residue was found in all the plaque proteins (fp-1 to fp-6) of mussels [58,63] and in the tubeworm proteins Pc-1 and Pc-2 [57]. 4-Hydroxyproline and 3,4-dihydroxyproline are the two hydroxylated derivatives of proline. They were detected in the decapeptide consensus repeat of the marine mussel protein Mefp-1 [3,129]. 4-Hydroxyarginine was found in the marine mussel protein Mefp-3 [91]. This protein has the ability to make conformational changes owing to its small size and the presence of glycine residues, a structural flexibility which enables the hydroxylated arginines to form hydrogen bonds with the substrate. 7-Hydroxytryptophan was found in the sequence of the protein Pvfp-1 from the mussel *P. viridis* [32]. In contrast to Mefp-1, only trace levels of DOPA were detected in Pvfp-1, what suggested the possibility that DOPA may be functionally replaced by hydroxytryptophan. Indeed, this modified amino acid resembles DOPA in having attributes that contribute to both cohesive and adsorptive interactions necessary for adhesion.

Below are some of the methods used to detect hydroxylation (mostly tyrosine hydroxylation) in bioadhesives.

*Amino acid analysis*. This method requires the hydrolysis of proteins, generally under acid conditions. Resulting amino acids are then separated by chromatography, detected and quantified by comparison with internal and external standards [130,131]. For instance, amino acid analysis allowed the highlighting of the presence of 3,4- and 4-hydroxyproline, and DOPA in mussel adhesive [132].

*Detection by staining*. Arnow staining specifically labels catechols, including DOPA [133]. The chemistry of the technique involves nitration of *o*-diphenols (catechols), resulting in a relatively stable diphenolate derivative with strong red colour. The nitroblue tetrazolium method indicates the presence of redox active compounds. It is based on the ability of DOPA-containing proteins to reduce nitroblue tetrazolium in the presence of alkaline pH and an excess of glycine (used as reducing agent). The resulting product, formazan, is a deep blue coloured compound [134]. Both methods were used to visualize the distribution of DOPA-containing proteins in the cement cells of the tubeworms *P. californica* [34,56] and *S. alveolata* [36]*.* The same methods can also be used on gels to stain DOPA-containing proteins after they have been separated by electrophoresis (e.g. [57,111]).

*Mass spectrometry*. MS is generally used in combination with a wide variety of separation methods (gel electrophoresis and chromatography techniques) to detect hydroxylation (see §3.2 for full explanation about MS). For instance, this methodology has been widely used to detect hydroxylation of the arginine residues in the mussel protein Mefp-3 [91].

*Quantitative UV spectroscopy*. The proportion of tyrosine residues modified to DOPA can be quantitatively measured by UV spectroscopy. The absorbencies of a 1 mM DOPA standard and of the DOPA-containing protein can be measured at 250–350 nm using a UV–visible spectrophotometer. The spectra differences can be determined by subtracting the spectra of the 1 mM DOPA standard from that obtained from the modified sample. Using the *λ*_max_ and Δ*ɛ* value of the 1 mM DOPA standard, the number of DOPA residues in the protein sample can be calculated according to the Lambert–Beer law [135].

#### 3.3.3. Phosphorylation

Protein phosphorylation is an important regulator of both cellular and extracellular events. Recently, protein phosphorylation has also emerged as an important process in biological adhesives. Among the four types of phosphorylation currently described (*O*-phosphorylation, *N*-phosphorylation, *S*-phosphorylation and acylphosphorylation) [136,137], only *O*-phosphorylation of serine residues has been detected in some marine adhesive proteins [87,120]. So far phosphoproteins have been reported among mussel foot proteins (Mefp-5 [29], Mgfp-5 [138], Mcfp-5 and Mcfp-6 [26]), and tubeworm cement proteins (Pc-3A and Pc-B [34]). In marine adhesives, phosphorylation is thought to impart a potential for both cohesive (by Ca^2+^ and Mg^2+^ bridging) and adhesive (as an adaptation for adhesion to calcareous substrata) contributions to the adhesive [26,53,84,118,139]. Moreover, phosphoserine (pSer) residues may also be involved in protein–protein cross-linking as they are thought to condense with histidine residues to form histidinoalanine cross-links with the loss of phosphate [140].

Different analytical methods have been developed for the detection and quantification of *O*-phosphorylation [136,137]. Below are some examples.

*Amino acid analysis*. The same procedure described for the detection of hydroxylation, although with some adjustment, can be applied to detect phosphoserine residues (e.g. in Mefp-5 [29]).

*Detection by staining*. Currently, fluorescent dyes are becoming the method of choice to stain phosphorylated proteins directly in acrylamide gels. The sensitivity is high (ng scale) and it can be combined with a total protein stain, allowing protein phosphorylation levels and expression levels to be monitored in the same gel. This approach was used by Santos *et al.* [109] to pinpoint putative adhesive proteins from the proteome of the tube feet in the sea urchin *P. lividus*. Within the total proteome, only 2% of the proteins were related with cell–cell or cell–substrate adhesion. Among those, one protein is homologous to nectin, a sea urchin secreted protein involved in embryonic cell–cell and cell–substrate adhesion. Two-dimensional gel staining with specific fluorescent stains revealed that in the tube foot nectin is present in eight isoforms of which five are simultaneously phosphorylated and glycosylated. Recent data confirmed that nectin is secreted in the sea urchin adhesive footprint (A. Toubarro *et al.* 2015, unpublished data). Although these fluorescent stains are optimized for staining sodium dodecylsulfate polyacrylamide gel electrophoresis gels, they can also be used in histochemistry on tissue sections, or on the secreted adhesive [141]. Nowadays, a large number of phospho-specific antibodies are commercially available, allowing pSer affinity-based staining. These antibodies can be used in western blot on extracted proteins and peptides, and also in immunohistochemistry on tissue sections. Antibodies can also be used to enrich and purify phosphorylated proteins and peptides. The use of an anti-pSer monoclonal antibody allowed the detection of phosphorylated proteins in the cement cells of the honeycomb worm *S. alveolata*, suggesting that, in this species, a polyphosphoprotein homologous to the protein Pc3 of *P. californica* would be also present [36,120].

*Mass spectrometry*. MS may be applied not only for detection of phosphorylation, but also for the identification of phosphorylation sites. Phosphorylation is detected by the analysis of mass spectra of trypsin-digested peptides that present a mass shift (*m*/*z* 79.9 or neutral loss *m*/*z* 80 or 98) in comparison with the theoretical peptide mass. This technique provides high speed and high sensitivity for detection of phosphorylation, but signals from phosphopeptides are generally weaker as compared with non-phosphorylated peptides and, therefore, it can be difficult to observe the signals from low-abundance phosphoproteins in the high background of abundant non-phosphorylated proteins. This can be overcome by enrichment of phophoproteins or phosphopeptides prior to MS. Waite & Qin [29] used MS to analyse purified Mefp-5 and observed peak intervals of about 80 Da suggestive of the presence of phosphate or sulfate groups. This was further confirmed by subjecting Mefp-5 to an alkaline phosphatase treatment that produced MS spectra with progressive mass decreases in decrements of 80–90 Da, which is the mass change associated with dephosphorylation.

### 3.4. Validation of the adhesive function of the protein

For a single organism or adhesive organ, the use of transcriptomics and proteomics can generate long lists of putative adhesive proteins (e.g. [101,109]). Not all these proteins are actually involved in the adhesion process and, after selection of an interesting candidate, it is important, therefore, to validate its adhesive function. This is usually done by confirming the localization of the protein in the adhesive cells as well as in the secreted adhesive, by knocking down the expression of the protein and evaluating the resulting phenotype, and/or by measuring the physico-chemical properties of recombinantly expressed proteins.

#### 3.4.1. Localization of the protein in adhesive cells and in the secreted adhesive

Genes and proteins identified by transcriptomics or proteomics need to be validated by corroborating their expression in the respective adhesive tissue or cell type.

Polyclonal antibodies represent a straightforward tool for detecting proteins in whole mount preparations or tissue sections [142,143]. They offer great sensitivity, are simple to handle, easy to store, and allow flexible applications (western blots, immunohistochemistry, immuno-TEM). Polyclonal antibodies are generated against one peptide or a complete protein. In general, the protein sequence (full-length or partial) is submitted to a commercial polyclonal antibody service provider. After bioinformatic analyses, a selection of 1–3 peptides is provided. These peptides will be chemically synthesized and used for immunization of two rabbits. Serum or affinity-purified antibodies will be delivered to the customer after about four months (but there are also rapid protocols). Disadvantages of polyclonal antibodies are the variability of different batches generated in different animals at different times, a higher potential for cross reactivity, and the limited amount of serum. Monoclonal antibodies, on the other hand, are quantitatively not restricted but require elaborate equipment and experience or are expensive when outsourced. Antibodies have been used in several model organisms to confirm the presence of putative adhesive proteins in the adhesive cells: e.g. the protein Sfp1 in sea stars [8], the polybasic proteins Pc-2, Pc-4 and Pc-5 in tubeworms [56], the protein Dpfp1 in zebra mussels [144], the proteins AgSF1 and AgSF2 in spiders [18] or the cement protein RIM36 in ticks [20]. In all these studies, the antibodies were also used to localize the proteins in the secreted adhesive, permitting one to investigate whether the protein is present at the interface (adhesive function) or in the bulk (cohesive function) of the adhesive layer.

If antibodies cannot be obtained (e.g. low immunogenicity of the protein like in the case of Pc-1 from tubeworms [56]), the localization of the corresponding mRNA can be visualized by *in situ* hybridization (ISH) [100,145]. By means of ISH a labelled RNA probe is produced and allowed to hybridize with the complementary mRNA. The labelled probe is then detected by antibody staining. In the flatworm *Macrostomum lignano*, ISH has been applied to study an adhesion-related intermediate-filament protein, macif1 [101]. Expression of the adhesive proteins Pc-1 to Pc-5 of the tubeworm *P. californica* has been studied by ISH, showing that some of these proteins are localized in cement cells with homogeneous granules while the others are localized in cement cells with heterogeneous granules [56]. Foot-specific expression of adhesion-related genes has also been demonstrated in both marine [146,147] and freshwater mussels [148,149].

#### 3.4.2. RNA interference

Understanding of the function of a gene or protein involved in bioadhesion is essential to elucidate the molecular foundation of the adhesion mechanism. RNA interference (RNAi) revolutionized the analysis of gene function by taking advantage of a cellular machinery that ultimately leads to the degradation of the mRNA of the gene of interest (e.g. [150,151]). Thereby, a loss-of-function phenotype is generated allowing conclusions to be drawn regarding the function of the target gene. The simplicity of application and the specificity of the knock-down by which the loss of gene function analysis can be achieved have boosted the use of RNAi in the past decade for model and non-model organisms. The RNAi mechanism is complex but today the components of the RNAi pathway are well understood [150,151]. Briefly, *in vitro* generated dsRNA molecules from 150 to 800 bp corresponding to the gene of interest are provided externally by soaking or are applied by injection. The dsRNA molecules become degraded into RNA duplexes of 21–25 bp in length by the endonuclease Dicer. The resulting short double stranded RNA molecules are called ‘small interfering RNAs’ (siRNAs). Next, Argonaute proteins bind and subsequently unwind the siRNA duplex into single strands. The antisense strand (the guiding strand) of the duplex becomes then incorporated into the RNA-induced silencing complex (RISC) while the other strand (the passenger strand) gets degraded. In the next step, messenger RNAs (mRNAs) with the complementary sequence to the RISC-located guiding strand are loaded into the RISC. Upon association between the two RNA molecules the mRNA gets cleaved. Eventually, this post-transcriptional gene silencing process results in the depletion of target mRNA molecules. Ultimately, as no new protein can be made, a knock-down phenotype of the respective transcript will be obtained. The lack of the particular protein will provide insight into its function. With respect to bioadhesion, the involved protein components could be functionally analysed, and their contribution to the adhesion process could be studied in detail with this method. In *M. lignano*, RNAi knock-down of *macif1* resulted in a lack-of-adhesion phenotype [101]. To the best of our knowledge, this is however the only study so far in which RNAi has been used to validate the adhesive function of a protein in metazoans.

#### 3.4.3. Recombinant proteins

To understand in detail the role of each protein component in a biological adhesive, it is necessary to obtain these components in large quantities. To bypass the problem of extracting adhesive proteins directly from animals, biomimetic molecules have been produced in the form of recombinant preparations of the adhesive proteins. This production has allowed the investigation of the essential functional characteristics of the proteins such as adsorption properties and adhesive properties on microscopic and macroscopic scales (e.g. [11,152,153]). Description of the methods allowing one to obtain these characteristics is out of the scope of this review but details can be found in [154,155].

To produce the heterologous adhesive proteins, different prokaryotic, eukaryotic and cell or tissue culture-based expression systems can be used. Common hosts include bacteria (*Escherichia coli*), yeasts (*Saccharomyces cerevisiae*), plants (tobacco) and mammals (goat, rabbit and mouse). Cell culture-based expression systems employ insect, plant and mammalian cells or tissue cultures instead of using the whole organism. All expression hosts have advantages and disadvantages that should be considered when making a choice. Some important variables for successful heterologous protein expression systems include: a codon usage compatible host; existence of promoter, transcriptional and translational regulators; optimal cultivation and protein purification methods; the ability to perform PTMs and to achieve correct folding of the recombinant protein; the possibility to provide high yields of the heterologous protein and easy scale-up process [27].

The recombinant DNA technology approach has been used mostly to produce large quantities of mussel adhesive proteins (fps) (see [42] for review). First attempts to obtain recombinant forms of the protein Mefp-1 started in the early 1990s [156,157]. However, these studies failed to obtain large amounts of recombinant proteins. In a later study, Lee *et al.* [158] expressed Mefp-1 fused to an *E. coli* signal peptide and thus optimized the expression of recombinant Mefp-1 in bacteria. Hwang *et al.* carried out two different studies with recombinant *M. galloprovincialis* foot protein 5 (rMgfp-5 [31]) and 3 (rMgfp-3 [152]). Both recombinant proteins were fused to a histidine tag and produced in the soluble fraction of *E. coli.* Even though recombinant mussel adhesive proteins were successfully produced and showed good performance in microscale adhesion tests, macroscale testing and large-scale applications were still not feasible. The main reasons included toxicity to the expression host, low expression levels and low solubility of the purified protein [27,159,160]. An additional problem related to the heterologous expression of adhesive proteins is that, generally, the recombinant proteins lack PTMs. These modifications include hydroxylation of proline, arginine and/or tyrosine residues. The production of functional recombinant mussel fps, therefore, requires an additional *in vitro* modification step: the enzyme-catalysed modification of tyrosine residues into DOPA. This is usually done using a commercially available mushroom tyrosinase [160].

In addition to mussel proteins, recombinant adhesive proteins were produced in *E. coli* from sequences obtained in barnacles, tubeworms, spiders and ticks. Two barnacle recombinant adhesive proteins, rMrcp-19k [11] and rMrcp-20k [15], have been successfully produced. The protein Sa-1 from the tubeworm *S. alveolata* was also successfully expressed and the recombinant protein, rSa-1, was mainly produced in the insoluble fraction of the bacteria, probably as inclusion bodies [59]. In spiders, fragments of the attachment disc glue silk fibroin 2 (PySp2) from *N. clavipes* [17] and the aggregate gland protein AgSF1 from *L. hesperus* [18] were produced and used for artificial spinning. Finally, in the tick *R. haemaphysaloides*, the cement protein RH50 was produced and used to immunize rabbits in order to challenge tick infestation [22].

## 4. Conclusion

Many metazoans rely on adhesive secretions to perform diverse functions. The diversity of biological adhesives is therefore huge and they can involve a large range of components with different functions and interactions. However, being mainly protein based, biological adhesives can be characterized by modern molecular tools, including the ‘omics' approach. In recent years, the combined use of transcriptomics and proteomics has emerged as the best way leading to the identification of novel adhesive proteins and retrieval of their complete sequences. For a single organism, however, the use of these tools can generate long lists of putative adhesive proteins which therefore require additional experiments to validate their function. When the extraction of adhesive molecules is hindered by the amount of tissue available or by the insolubility of the material, alternative molecular tools exist to circumvent the problems at different levels from genes to secreted proteins. The wide range of experimental strategies compiled in this review are useful not only for natural adhesives but also for other type of biological materials.

## Endnote

1 Polyphenol oxidases catalyse the *o*-hydroxylation of monophenols (e.g. tyrosine) to *o*-diphenols (e.g. DOPA) and the further oxidation of *o*-diphenols to *o*-quinones. Enzyme nomenclature differentiates between monophenol monooxygenases, traditionally called tyrosinases (EC 1.14.18.1), and catechol oxidases (EC 1.10.3.1). Tyrosinases can oxidize tyrosine to l-DOPA and l-DOPA to *o*-quinone. By contrast, catechol oxidases oxidize l-DOPA to *o*-quinone. The formation of *o*-quinone is responsible for the browning/tanning/curing processes that occur in plants, bacteria and animals [161].
